# The co-occurrence of adverse childhood experiences and mental health among Latina/o adults: A latent class analysis approach

**DOI:** 10.1016/j.pmedr.2023.102185

**Published:** 2023-03-30

**Authors:** Michael Niño, Kazumi Tsuchiya, Shaun Thomas, Christian Vazquez

**Affiliations:** a Department of Sociology and Criminology, University of Arkansas, 1 University of Arkansas, Fayetteville, AR 72701, USA; b Dalla Lana Social of Public Health, University of Toronto, 27 Kings College Circle, Toronto, Ontario M5S 1A1, Canada; c School of Social Work, The University of Texas at Arlington, 211 S. Cooper, Arlington, TX 76019, USA

**Keywords:** Adverse childhood experiences, Mental health, Latent class analysis, Depressive symptoms, Latino mental health

## Abstract

Adverse childhood experiences (ACEs) have been linked to poor mental health among Latina/os. Few studies, however, have attempted to understand the extent to which ACEs co-occur and whether different forms of ACE co-occurrence differentially shape poor mental health patterns among Latina/os. The present study begins to address this gap by (1) identifying latent classes of ACEs and (2) determining whether and how different ACE classes shape high depressive symptoms among Latina/o adults. Data were drawn from two waves of the Hispanic Community Health Study/Study of Latinos, a longitudinal, community-based sample of Latina/os living in four urban communities. Latent Class Analysis (LCA) was used to identify subgroups of Latina/os who were exposed to co-occurring forms of maltreatment. Results from the LCA revealed four classes: (1) high ACEs, (2) emotional and physical abuse, (3) low ACEs, and (4) household alcohol/drug use and parental separation/divorce. Regression analyses indicate, when compared to the low ACEs class, Latina/os in the high ACEs class and emotional/physical abuse class were more likely to report high depressive symptoms. Findings from this study demonstrate ACEs co-occur in distinct classes of maltreatment and different combinations of ACEs uniquely shape the risk of poor mental health among Latina/os. Results from this study can help inform tailored mental health interventions for Latina/os that have a history of ACE exposure.

## 1 Introduction

Adverse childhood experiences (ACEs) encompass an array of stressful and/or traumatic life events that occur before the age of 18 and include, but are not limited to, various types of physical, emotional, and sexual abuse and household dysfunction. Scholarship finds ACEs have been associated with a range of poor mental health outcomes in adulthood including depression (Chapman et al., 2004, Mwachofi et al., 2020), anxiety (Reiser et al., 2014, Sareen et al., 2013), antisocial behavior (Schilling et al., 2007) and suicidality (Dube et al., 2001).

Given the significant mental health consequences associated with ACEs, a small but growing body of literature has sought to better understand the potential linkages between ACEs and mental health among one of the fast-growing racial and ethnic groups in the U.S., Latina/os. While research focusing on ACEs among Latina/os is limited, scholars find most Latina/os have been exposed to at least one ACE, and more than a quarter have experienced four or more ACEs (Llabre et al., 2017). Concerning mental health, emerging research finds ACEs increase the risk of depressive symptoms among Latina/os, generally, and across a myriad of Latina/o subpopulations. For instance, ACEs have been linked to depressive symptoms for Latina/o migrant farmworkers (Andrews et al., 2020), as well as for Latina/os living in rural (Barrera et al., 2019) and urban settings (Llabre et al., 2017). Similarly, among Latina/o youth, ACEs have been associated with emotional dysregulation (Zetino et al., 2020).

Despite evidence of negative mental health consequences associated with ACEs for Latina/os, several gaps remain. First, little scholarship has attempted to identify distinct ACE categories among Latina/os or assess whether and how such categories may differentially shape mental health patterns. Most scholarship examining mental health consequences associated with ACEs uses a cumulative or count-based approach when attempting to assess ACE exposure (Anda et al., 2007, Dong et al., 2004, Dube et al., 2001). This approach is guided by evidence showing an increase in ACE exposure is significantly associated with a rise in poor mental health (Anda et al., 2007, Chapman et al., 2004, Grigsby et al., 2020).

Although the cumulative approach has provided interesting insights, an emerging body of work argues the approach treats ACEs equally and is unable to determine whether a particular set of ACEs co-occur and whether certain forms of ACE co-occurrence pose more of a risk to mental health than other forms. Guided by the proposition that specific ACEs and/or a combination of ACE exposures potentially pose different health risks, a promising body of research has begun to identify and test whether unique, co-occurring, ACE combinations differentially shape health and wellbeing patterns.

To address limitations tied to the cumulative-based approach, scholars have taken advantage of statistical techniques, such as latent class analysis (LCA), to empirically identify clusters of respondents with similar ACEs. Identifying subgroups of maltreatment has been a focus of research for many years, given the identification and understanding of subgroups can lead to more targeted policy and practitioner-based interventions (Lanza and Rhoades, 2013, Tomczyk et al., 2016). Within this line of work, scholars have demonstrated that ACE exposures co-occur and that different combinations of co-occurring forms of ACEs increase the risk of a myriad of poor physical and mental health outcomes (Brown et al., 2019, Lanier et al., 2018, Schilling et al., 2007, Shin et al., 2018).

To our knowledge, only one study to date has attempted to identify co-occurring ACE exposures among Latina/os. Using a clinical sample of Latinas, Alvarez et al. (2022) identified five ACE categories: (1) global ACEs cluster, (2) collective and community violence, (3) high physical and emotional abuse, (4) household dysfunction with physical and emotional abuse, and (5) low ACEs. The authors also found, when compared to respondents with low ACEs, Latinas exposed to community violence, physical abuse, and a multitude of ACEs were at a greater risk of depressive symptoms, anxiety, and PTSD symptoms.

One limitation in that study, along with most Latina/o ACEs-mental health scholarship, is the use of cross-sectional data. Scholars have yet to determine whether previously documented deleterious mental health consequences of ACEs remain when utilizing longitudinal frameworks. More research is needed leveraging data that provides scholars with the ability to address concerns related to the timing of ACEs and mental health. The use of such data can provide scholars with new insights into how ACEs shape mental health patterns among Latina/os over time.

Using two waves from the Hispanic Community Health Study/Study of Latina/os (HCHS/SOL), the following study attempts to expand our understanding of the complex relationship between ACEs and mental health by first identifying whether and how ACEs co-occur among Latina/o adults using LCA. To understand the long-term impact of ACEs on mental health among Latina/os, we then investigate whether different classes of ACEs identified at Time 1 significantly shape depressive symptoms at Time 2. Better understanding whether and how co-occurring ACEs shape mental health patterns can aid policymakers and clinicians in developing more effective interventions designed to reduce the harmful long-term mental health consequences tied to child maltreatment.

## 2 Methods

### 2.1 Participants and data collection

The HCHS/SOL is a longitudinal, community-based study of Latina/os from the following ethnic backgrounds: Cuban, Puerto Rican, Dominican, Mexican, and South/Central American. Respondents were selected from four stable Latina/o communities (Bronx, Chicago, Miami, and San Diego) from 2008 to 11. The baseline HCHS/SOL survey, which occurred between March 2008 and June 2011, examined 16,623 self-identified Latina/os from the four specified communities.

In 2009, a subsample of 5,313 HCHS/SOL respondents participated in the Sociocultural Ancillary Study, which includes measures that capture a range of socioeconomic and cultural mechanisms, social and psychological processes, and life course events. From 2014 to 2017, a follow-up examination, known as Visit 2, captured survey responses and biomarker and anthropometric data from 11,623 Latina/os that participated in the baseline HCHS/SOL survey. For the current study, data were drawn from a sample of 3,251 Latina/o adults that participated in both the Sociocultural Ancillary Study (Time 1; 2009) and Visit 2 (Time 2; 2014–2017) of the HCHS/SOL. For inclusion in the final analytic sample, respondents must have had valid responses for all measures and valid sampling weights. The final analytic sample included 2,586 Latina/os from four urban communities. Most of the attrition in the final analytic sample size was due to missing sampling weights (95 percent of cases were due to omitted weights, while the remaining 5 percent were mostly due to missing responses tied to annual household income and educational status). Institutional Review Board approval was obtained from all study sites for all HCHS/SOL study procedures and materials, and all participants provided written informed consent. This study was also approved by the Institutional Review Board at the University of Arkansas, Fayetteville.

### 2.2 Measures

#### 2.2.1 Depressive symptoms

Depressive symptoms was assessed using the abbreviated 10-item Center for Epidemiological Studies of Depression (CES-D). Original responses ranged from 0 (*rarely or none of the time*) to 3 (*all of the time*) and captured a series of possible symptoms such as “I was bothered by things that usually don’t bother me”, “I felt lonely”, and “I had trouble keeping my mind on what I was doing”. Scores on the overall CES-D ranged from 0 to 30 and were dichotomized to capture respondents exhibiting “high depressive symptoms”. Specifically, CES-D scores >= 10 were categorized as “high depressive symptoms”, indicating evidence of clinically significant symptoms of depression. It is important to note that this categorization is not equivalent to a clinical diagnosis of major depression. Prior studies have demonstrated good sensitivity and specificity with the “high depressive symptoms” cut-off point for the 10-item CES-D scale (Andresen et al., 1994) and this cutoff has been used in similar HCHS/SOL studies (McCurley et al., 2019). Internal consistency and reliability for the 10-item CES-D was α = 0.82 for both the English and Spanish surveys.

#### 2.2.2 Adverse childhood experiences

Exposure to ACEs was assessed using 10 items drawn from a study led by the Centers for Disease Control and Kaiser Permanente focused on understanding the role childhood maltreatment plays in shaping health and well-being in later life (Felitti et al., 1998). Table 1 provides a detailed description of each item.

**Table 1:** Proportion of Latina/o adults with Adverse Childhood experiences.

**Variable name**	**Description of survey ACE items**	**Proportion**
Swear	*Did a parent or other adults in the household often or very often swear at you, insult you, put you down or humiliate you? Or act in a way that made you afraid that you might by physically hurt?*	0.31
Parent push	*Did a parent or other adult in the household often or very often push, grab, slap, or throw something at you or ever hit you so hard that you had marks or were injured?*	0.29
Touch	*Did an adult or person at least 5 years older than you ever touch or fondle you or have you touch their body in a sexual way or attempt or actually have oral, anal, or vaginal intercourse with you?*	0.16
Love	*Did you often or very often feel that no one in your family loved you or thought you were important or special or Your family didn’t look out for each other, feel close to each other, or support each other?*	0.23
Eat	*Did you often or very often feel that you didn’t have enough to eat, had to wear dirty clothes, and had no one to protect you or your parents were too drunk or high to take care of you or take you to the doctor if you needed it?*	0.12
Divorce	*Were your parents ever separated or divorced?*	0.41
Mom push	*Was your mother or stepmother: Often or very often pushed, grabbed, slapped, or had something thrown at her? Or Sometimes, often, or very often kicked, bitten, hit with a fist, or hit with something hard? Or Ever repeatedly hit at least a few minutes or threatened with a gun or knife?*	0.19
Alcohol	*Did you live with anyone who was a problem drinker or alcoholic or who used street drugs?*	0.30
Depression	*Was a household member depressed or mentally ill, or did a household member attempt suicide?*	0.20
Prison	*Did a household member go to prison?*	0.24
Cumulative ACE score		2.47(0.2.31)

#### 2.2.3 Covariates

Covariates include ethnic background (Dominican, Cuban, Puerto Rican, Central/South American, and Mexican), gender, age, educational attainment (elementary/primary school, middle school/junior high, high school/prep school), nativity (U.S. born, foreign-born), marital status (married, not married), homeownership (homeowner, not a homeowner), employment status (unemployed, employed part-time, employed full-time), household income (<$10,000, $10,001–$20,000, $20,001–29,999, $30,000–$40,000, more than $40,000), language acculturation, social acculturation, and familism. Language and social acculturation were derived from the Short Acculturation Scale for Hispanics (Marin et al. 1987). Familism was measured using the 14-item Familism scale (Sabogal et al., 1987).

### 2.3 Analytic strategy

Our analysis unfolds by, first, performing LCA on all 10 dichotomous ACE items. For each model, we assessed the concern of multiple modes by generating ten sets of random starting values. If at least 80% of sets converged to the same solution, we assumed the model was identified. If less than 80% of sets converged, 100 random starting values were used to determine the confidence with which the maximum likelihood solution could be identified. To determine the best-fitting model, an iterative process was used. The optimal class solution was selected based on the following fit indices: (1) Bayesian Information Criterion (BIC), (2) Akaike Information Criterion (AIC), (3) Lo-Mendell-Rubin adjusted likelihood ratio test (LMRT), and (4) bootstrap likelihood ratio test (BLRT). In addition to the model fit statistics, we considered the theoretical meaningfulness of the classes, whether results were interpretable, and the percentage of respondents represented in each class. Model precision for classification was evaluated using relative entropy values. Entropy values closer to 1.00 indicate better precision.

We then provide descriptive statistics of sample characteristics for the total sample and across ACE classes. To test whether there were significant differences in high depression, and other covariates by ACE classes, tests of differences were included. Logistic regression models were used to produce odds ratios (ORs) to assess relationships between ACE classes and high depressive symptoms for Latina/o adults. In the baseline or “unadjusted” model, we examine bivariate associations between ACE classes and high depressive symptoms. In the subsequent model, we adjust for all specified covariates to isolate the role of different ACE classes on high depressive symptoms from other factors that may influence depressive symptoms. Analyses adjusted for the clustered nature of HCHS/SOL design. To account for the unequal probability of selection due to oversampling of Latina/os aged 45–74, analyses were weighted using post-stratification weights.

## 3 Results

### 3.1 Adults with ACEs

Table 1 provides a detailed description, along with the corresponding proportion of respondents exposed to each ACE included in this study. Results demonstrate the most common ACE among respondents was parental separation and divorce (41%). We also find that approximately 1 out of 3 respondents were exposed to emotional abuse (31%), household physical abuse (29%), and household alcohol and drug abuse (30%). Finally, on average, findings indicate that Latina/o adults were exposed to 2.47 ACEs.

### 3.2 Latent classes of ACEs

Table 2 provides model fit indices for the 2-class through 5-class models. Based on the various fit indices and selection criteria, the 4-class solution was determined to be the best fitting and most parsimonious model. Although the 5-class solution provided marginally lower AIC and BIC values compared to the 4-class solution, LMRT indicated that the 5-class solution was not significantly better than the prior solution. Moreover, entropy scores for the 4-class solution exceeded the recommended value of 0.70 and the solution was closer to 1 (0.71) when compared to the 5-class solution, indicating slightly better precision in class prediction. Finally, less than 5 percent of respondents were placed into the fifth class, which may result in less reliable estimates of class-specific parameters and obfuscate the substantive meaning of the class solutions.

**Table 2:** Fit indices for latent class analysis for 1–5 classes.

	Class 2	Class 3	Class 4	Class 5
Model fit indices				
Log-likelihood	−12479.426	−12343.52	−12245.312	−12219.244
Entropy	0.827	0.687	0.71	0.70
AIC	25000.852	24751.04	24576.625	24546.489
Adjusted BIC	25057.161	24836.843	24691.923	24691.282
LMR p-value	<0.001	<0.001	<0.001	0.313
BLRT *p*-value	<0.001	<0.001	<0.001	<0.001

*Note:* AIC = Akaike information criterion; Adjusted BIC = sample-size adjusted Bayesian information criterion; LMR *p-*value = Lo-Mendell-Rubin adjusted likelihood ratio test; BLRT *p*-value bootstrap likelihood ratio test.

**Fig. 1:**
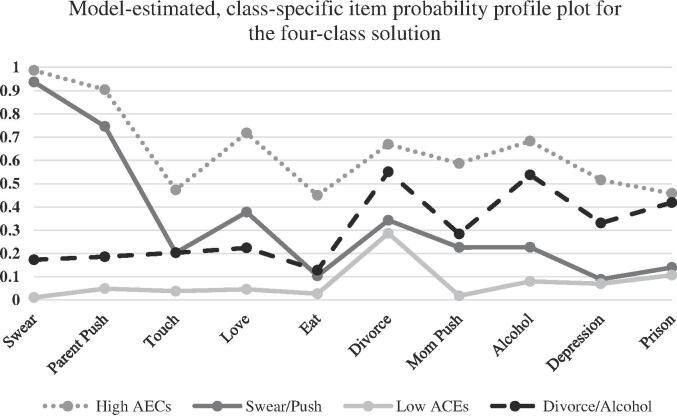
Proportion of Latina/os with adverse childhood experiences for the four-class solution, *Note:* High ACEs = high ACEs class *(class 1),* Swear/Push = emotional and physical abuse class *(class 2)*; low ACEs = low ACEs class (*class 3*); Divorce/Alcohol = household alcohol and drug use parental separation and divorce class *(class 4).*

Fig. 1 provides the item-response probabilities for the 10 specific ACEs across each of the 4 classes. The four classes and their respective names and the percentage of respondents in each respective class are provided in Table 3: Low ACEs exposure (53%), Divorce/Alcohol (22%), High ACEs exposure (15%), and Swear/Push (10%). Fig. 1 demonstrates that participants in the high ACEs class had the highest probability of endorsing the 10 ACEs included in this study. More specifically, respondents in this class exhibited high to moderate (0.99–0.070) probabilities of exposure to emotional abuse (0.99), physical abuse (0.90), and neglect (0.70). Respondents in this class also exhibited moderate (0.68–0.45) probabilities for exposure to household alcohol and drug use, divorce, maternal physical abuse, household mental illness, sexual abuse, and imprisonment of a household member. The emotional and physical abuse class was differentiated from the other classes by exhibiting high probabilities of emotional (0.94) and physical abuse (0.75) but low probabilities for the remaining ACEs exposures (0.37–0.09). Participants in the low ACEs class had low probabilities (0.28–0.01) of exposure to all 10 ACEs included in this study. In the final class, respondents exhibited moderate probabilities for exposure to household alcohol and drug use (0.54) as well as parental separation and divorce (0.55), however, they exhibit substantively lower scores on all other ACEs (<0.40), except for imprisonment of a household member.

**Table 3:** Descriptive statistics for the overall sample and by ACE classes.

	**Overall**	**High ACEs**	**Swear/Push**	**Low ACEs**	**Divorce/Alcohol/Separation**
	Mean(S.D)	Mean(S.D)	Mean(S.D)	Mean(S.D)	Mean(S.D)
High depression (Score >=10)	0.24(0.01)	0.34(0.03)	0.30(0.04)	0.19(0.02)	0.25(0.03)***
*Latent classes*					
High AECs exposure	0.15(0.01)				
Swear/Push	0.10(0.01)				
Low ACEs exposure	0.53(0.01)				
Divorce/Alcohol/Separation	0.22(0.01)				
*Ethnic Background*					
Dominican	0.11(0.01)	0.07(0.02)	0.10(0.02)	0.12(0.01)	0.10(0.02)***
Central/South American	0.12(0.01)	0.10(0.02)	0.16(0.03)	0.13(0.01)	0.08(0.01)
Cuban	0.22(0.01)	0.19(0.03)	0.12(0.03)	0.27(0.02)	0.20(0.03)
Mexican	0.36(0.01)	0.36(0.04)	0.46(0.04)	0.33(0.02)	0.38(0.03)
Puerto Rican	0.19(0.01)	0.27(0.03)	0.16(0.03)	0.15(0.01)	0.24(0.03)
*Gender*					
Male	0.52(0.01)	0.41(0.04)	0.57(0.04)	0.55(0.02)	0.50(0.03)**
Age	42.73(0.44)	42.42(1.13)	42.17(1.17)	43.28(0.62)	41.86(0.94)
*Education*					
Elementary primary	0.08(0.01)	0.11(0.03)	0.10(0.02)	0.06(0.01)	0.08(0.02)
Middle school/junior high	0.09(0.01)	0.10(0.02)	0.08(0.02)	0.09(0.01)	0.09(0.02)
High school/prep school	0.43(0.01)	0.45(0.04)	0.43(0.04)	0.41(0.02)	0.45(0.03)
Trade school/vocational	0.12(0.01)	0.14(0.03)	0.12(0.03)	0.11(0.01)	0.13(0.02)
University/college	0.28(0.01)	0.21(0.03)	0.27(0.04)	0.32(0.02)	0.25(0.03)
*Nativity*					
U.S. born	0.32(0.01)	0.38(0.04)	0.27(0.04)	0.28(0.02)	0.39(0.03)**
*Marital status*					
Not married	0.52(0.01)	0.50(0.04)	0.49(0.04)	0.51(0.02)	0.55(0.03)
Homeowner	0.24(0.01)	0.23(0.03)	0.26(0.04)	0.25(0.02)	0.21(0.02)
Employment Status					
Unemployed	0.52(0.01)	0.55(0.04)	0.50(0.04)	0.52(0.02)	0.53(0.03)
Employed part-time	0.20(0.01)	0.22(0.03)	0.25(0.04)	0.17(0.02)	0.22(0.03)
Employed full-time	0.28(0.01)	0.23(0.03)	0.25(0.03)	0.31(0.02)	0.25(0.03)
Household income					
Less than $10,000	0.18(0.01)	0.25(0.03)	0.15(0.03)	0.17(0.02)	0.17(0.02)**
$10,001-$20,000	0.33(0.01)	0.35(0.04)	0.31(0.04)	0.30(0.02)	0.39(0.03)
$20,001–29,999	0.20(0.01)	0.19(0.02)	0.20(0.03)	0.22(0.02)	0.17(0.02)
$30,000-$40,000	0.13(0.01)	0.09(0.02)	0.12(0.03)	0.14(0.01)	0.12(0.02)
More than $40,000	0.17(0.01)	0.12(0.02)	0.22(0.04)	0.18(0.02)	0.14(0.02)
Acculturation					
Language acculturation	2.11(0.03)	2.21(0.08)	2.16(0.09)	2.01(0.05)	2.27(0.08)
Social acculturation	2.25(0.02)	2.29(0.04)	2.30(0.05)	2.22(0.02)	2.29(0.04)
Familism	3.62(0.02)	3.58(0.04)	3.56(0.05)	3.66(0.02)	3.60(0.03)
	*N* = 2586	*N* = 404	N = 293	N = 1356	N = 533

*Data:* Hispanic Community Health Study/Study of Latinos (Time 1 2009-Time 2 2014–2017).

Asterisks indicate significant differences between ACEs classes. *p <.05, **p <.01, ***p <.001. Descriptive statistics were weighted and adjust for the complex design of the HCHS/SOL.

### 3.3 Descriptive statistics for the overall sample and by ACE class

Table 3 provides weighted descriptive statistics for the overall sample and across ACE categories. Results from the overall sample indicate that almost one-fourth (24%) of respondents exhibited high depressive symptoms. The majority of the overall sample were male (52%), not married (52%), unemployed (52%), and report $20,000 or less in household income (51%). Results from Table 3 also demonstrate notable differences in high depressive symptoms across ACE classes. Specifically, respondents in the high ACEs class were more likely to report high depressive symptoms (34%) relative to each of the other ACE classes (swear/push 30%, low ACEs 19%, divorce/alcohol 25%). We also find Latino males (41%) are least likely to be in the high ACEs class relative to their female counterparts (59%). Moreover, respondents born in the U.S. were relatively more likely to be in the high ACEs and the divorce/alcohol class. Interestingly, education level, marital status, employment status, homeownership, and measures of acculturation do not vary substantively across ACE classes.

### 3.4 Multivariate regression results

Table 4 contains survey-corrected logistic regression estimates that assess the relationship between ACE classes and high depressive symptoms. Results from model 1, the “unadjusted model”, demonstrate that ACE classes play a significant role in high depressive symptoms among Latina/o adults. When compared to the low ACEs class, results indicate that respondents in the high ACEs class (OR = 2.25, 95% CI = 1.59–3.18), emotional and physical abuse class (OR = 1.85, 95% CI = 1.22–281), and household alcohol/drug use and parental separation class (OR = 1.43, 95% CI = 1.04–1.98) were statistically significantly more likely to report high depressive symptoms. In model 2, we adjust for a range of additional social, cultural, and demographic characteristics. Results from model 2 demonstrate similar patterns to the “unadjusted model”, indicating some ACE classes are significantly associated with high depressive symptoms even after controlling for a host of additional factors. For instance, compared to the low ACEs class, we continued to observe that the odds of reporting high depressive symptoms were significantly greater for the high ACEs class (OR = 1.95, 95% CI = 1.40–2.73) and physical abuse class (OR = 2.02, 95% CI = 1.28–3.20). After accounting for other covariates, however, results also indicate that exposure to household alcohol/drug use and parental separation no longer significantly increased the risk of high depressive symptoms relative to the low ACEs class.

**Table 4:** Estimated associations between ACE classes and high depressive symptoms.

	Model 1	Model 2
	OR[ 95 %CI]	AOR[95 %CI]
Constant	0.24*** [0.20–0.29]	0.03***[0.01–0.10]
*Latent classes*		
High ACEs exposure	2.25** [1.59–3.18]	1.95***[1.40–2.73]
Swear/push	1.85** [1.22–2.81]	2.02***[1.28–3.20]
Divorce/alcohol/separation	1.43** [1.04–1.98]	1.31 [0.92–1.85]
*Ethnic background*		
Dominican		1.96** [1.19–3.43]
Central/South American		1.29 [0.82–2.02]
Cuban		1.40 [0.93–2.11]
Puerto Rican		2.03* [1.06–3.87]
*Gender*		
Male		0.60***[0.46–0.77]
Age		1.01** [1.00–1.03]
*Education*		
Elementary/primary		1.41 [0.81–2.43]
Middle school/junior high		2.17***[1.38–3.42]
Trade school/vocational		1.00 [0.66–1.54]
University/college		0.69* [0.49–0.99]
*Nativity*		
U.S. born		0.77 [0.77–0.23]
*Marital status*		
Not married		1.30 + [0.99–1.72]
Homeowner		0.77 [0.53–1.11]
*Employment status*		
Unemployed		1.97***[1.42–2.73]
Employed part-time		1.26 [0.78–2.04]
*Household income*		
Less than $10,000		1.16 [0.63–2.12]
$10,001-$20,000		1.21 [0.69–2.12]
$20,001–29,999		0.87 [0.49–1.54]
$30,000-$40,000		0.68 [0.39–1.18]
*Acculturation*		
Language acculturation		1.29** [1.07–1.56]
Social acculturation		1.04 [0.78–1.38]
Familism		1.12 [0.88–1.42]
	*N =* 2586	*N =* 2586

*Data:* Hispanic Community Health Study/Study of Latinos (Time 1 2009-Time 2 2014–2017).

*Note:* OR = odds ratio; CI = confidence internal; AOR = adjusted odds ratio.

*+p < 0*.10, *p <.05, **p <.01, ***p <.001.

## 4 Discussion

Consistent with prior research, we find evidence that Latina/os are exposed to a multitude of ACEs and, in many cases, ACE exposures co-occur. At baseline, findings from our descriptive analyses demonstrated, on average, Latina/o adults reported experiencing 2.47 ACEs and that parental separation and divorce were the most common forms of ACE exposure. Building on the results from our descriptive analyses, LCA estimates revealed multiple classes of Latina/o adults that can be distinguished by different patterns of ACEs exposure. More specifically, model fit indices from our LCA indicate that the four-class solution was the best-fitting model. The four classes were labeled as follows: (1) high ACEs, (2) emotional and physical abuse, (3) low ACEs, and (4) parental separation and divorce.

Largely, our findings are consistent with a growing number of studies that provide empirical evidence of distinguishable classes of ACEs among adults, including the lone clinical study focused on Latinas (Alvarez et al., 2022, Lee et al., 2020, Shin et al., 2018). More specifically, similar studies offer empirical evidence of multiple ACE classes that range from high ACE exposure to low ACE exposure. Moreover, the emotional/physical abuse and parental separation/divorce classes have also been identified in other ACE studies focused on the general U.S. population (Maxia, et al., 2004). Taken as a whole, findings from our LCA support the limited existing evidence that ACE exposures co-occur among Latina/os and that such co-occurrence differentially impacts depressive symptoms. For scholars attempting to better understand the complex nature of ACE exposures among Latina/os, findings from this study demonstrate that LCA can be a useful tool for identifying distinct co-occurring forms of childhood adversity among this growing but often understudied population in the U.S. Moreover, using person-centered approaches, such as LCA, we are better able to understand differential risks tied to ACE types, which gives scholars and practitioners the ability to tailor support and interventions for each observed ACE subgroup.

When examining whether ACE classes differentially shape mental health patterns, results demonstrate important linkages between co-occurring ACE exposures at time 1 and high depressive symptoms among Latina/o adults at time 2. For instance, in the baseline model, when compared to the low ACE exposure class, we observed an increased risk of high depressive symptoms for all three of the other distinguishable ACE classes. Importantly, after accounting for covariates, we find the increased risk of high depressive symptoms for the household alcohol/drug and parental separation/divorce class relative to the low ACEs class was no longer statistically significant. We did, however, continue to observe a significantly greater likelihood of high depressive symptoms among Latina/o adults in the high ACEs class and emotional/physical abuse class in comparison to the low ACEs class.

Overall, findings from the current study contribute to the extant literature in demonstrating that, after accounting for important covariates tied to maltreatment and depression, high exposure to multiple forms of childhood adversities significantly increases the risk of poor mental health among Latina/os. These findings align with the limited extant scholarship that suggests Latina/o adults with more exposure to ACEs are at a greater risk for depressive symptoms (Alvarez et al., 2022). Our results also indicate that exposure to physical and emotional abuse from someone within the household can have long-term mental health consequences for Latina/os in adulthood. As mentioned above, our results provide valuable direction for policymakers and clinicians invested in developing interventions designed to reduce mental health disparities for Latina/o adults with a history of ACEs. Specifically, our findings point to the need to tailor interventions for specific patterns of co-occurring maltreatment, such as those with a history of physical and emotional abuse or high exposure to multiple ACEs.

Although the current study offers substantive contributions to the extant literature, certain limitations should be noted and potentially addressed in future research. First, although the validity and reliability of the ACE items used have been established in prior research (Hardt and Rutter, 2004), it is important to note that the retrospective nature of ACE questions could be subject to recall bias. Moreover, while the ACE items included in this study have been widely used in child maltreatment work, some scholars argue that the singular focus on at-home childhood adversities limits our understanding of the broad consequences associated with adversities tied to socio-structural conditions, in particular, for historically marginalized groups (Oh et al., 2018). For instance, current ACE frameworks have yet to account for psychological and structural violence exposures tied to restrictive immigration policies and practices that disproportionately impact Latina/o communities. Future ACE scholarship focused on mental health outcomes could benefit greatly from emerging ecological frameworks that capture childhood adversities tied to deprivation, detention, and deportation for Latina/os that were born outside the United States (Barajas-Gonzalez et al., 2021).

Finally, the HCHS/SOL sampling design focuses on four urban centers in the U.S., therefore we are unable to generalize our findings to Latina/os living in rural and suburban areas. Moreover, given that the sample was majority male, unmarried and unemployed, future ACE studies focused on Latina/os would benefit from sampling strategies that capture responses from a nationally representative sample, so that we can better understand whether the observed patterns in this study remain after accounting for important structurally rooted mechanisms such as gender, marital status, and employment status.

## 5 Contributions to the manuscript

*Dr. Niño:* study design, project development, data management and analysis, manuscript writing/editing, and overall supervision of the project.

*Dr. Tsuchiya:* study design, manuscript writing/editing.

*Dr. Thomas:* study design, manuscript writing/editing.

*Dr. Vasquez:* study design, manuscript writing/editing.

## Declaration of Competing Interest

The authors declare that they have no known competing financial interests or personal relationships that could have appeared to influence the work reported in this paper.

## Data Availability

The authors do not have permission to share data.

## References

[b0005] Alvarez C., Sabina C., Brockie T., Perrin N., Sanchez-Roman M.J., Escobar-Acosta L., Vrany E., Cooper L.A., Hill-Briggs F. (2022). Patterns of adverse childhood experiences, social problem-solving, and mental health among Latina immigrants. J. Interpersonal Violence.

[b0010] Anda R.F., Brown D.W., Felitti V.J., Bremner J.D., Dube S.R., Giles W.H. (2007). Adverse childhood experiences and prescribed psychotropic medications in adults. Am. J. Prev. Med..

[b0015] Andresen E.M., Malmgren J.A., Carter W.B., Patrick D.L. (1994). Screening for depression in well older adults: evaluation of a short form of the CES-D. Am. J. Prev. Med..

[b0020] Andrews A.R., Haws J.K., Acosta L.M., Acosta Canchila M.N., Carlo G., Grant K.M., Ramos A.K. (2020). Combinatorial effects of discrimination, legal status fears, adverse childhood experiences, and harsh working conditions among Latino migrant farmworkers: Testing learned helplessness hypotheses. J. Latinx Psychol..

[b0025] Barajas-Gonzalez R.G., Ayón C., Brabeck K., Rojas-Flores L., Valdez C.R. (2021). An ecological expansion of the adverse childhood experiences (ACEs) framework to include threat and deprivation associated with US immigration policies and enforcement practices: An examination of the Latinx immigrant experience. Soc. Sci. Med..

[b0030] Barrera I., Sharma V., Aratani Y. (2019). The prevalence of mental illness and substance abuse among rural Latino adults with multiple adverse childhood experiences in California. J. Immigr. Minor. Health.

[b0035] Brown S.M., Rienks S., McCrae J.S., Watamura S.E. (2019). The co-occurrence of adverse childhood experiences among children investigated for child maltreatment: A latent class analysis. Child Abuse Negl..

[b0040] Chapman D.P., Whitfield C.L., Felitti V.J., Dube S.R., Edwards V.J., Anda R.F. (2004). Adverse childhood experiences and the risk of depressive disorders in adulthood. J. Affect. Disord..

[b0045] Dong M., Giles W.H., Felitti V.J., Dube S.R., Williams J.E., Chapman D.P., Anda R.F. (2004). Insights into causal pathways for ischemic heart disease: adverse childhood experiences study. Circulation.

[b0050] Dube S.R., Anda R.F., Felitti V.J., Chapman D.P., Williamson D.F., Giles W.H. (2001). Childhood abuse, household dysfunction, and the risk of attempted suicide throughout the life span: findings from the Adverse Childhood Experiences Study. JAMA.

[b0055] Felitti V.J., Anda R.F., Nordenberg D., Williamson D.F., Spitz A.M., Edwards V., Mark J.S. (1998). Relationship of childhood abuse and household dysfunction to many of the leading causes of death in adults: The Adverse Childhood Experiences (ACE) Study. Am. J. Prev. Med..

[b0060] Grigsby T.J., Rogers C.J., Albers L.D., Benjamin S.M., Lust K., Eisenberg M.E., Forster M. (2020). Adverse childhood experiences and health indicators in a young adult, college student sample: Differences by gender. Int. J. Behav. Med..

[b0065] Hardt J., Rutter M. (2004). Validity of adult retrospective reports of adverse childhood experiences: review of the evidence. J. Child Psychol. Psychiatry.

[b0070] Lanier P., Maguire-Jack K., Lombardi B., Frey J., Rose R.A. (2018). Adverse childhood experiences and child health outcomes: comparing cumulative risk and latent class approaches. Matern. Child Health J..

[b0075] Lanza S.T., Rhoades B.L. (2013). Latent class analysis: an alternative perspective on subgroup analysis in prevention and treatment. Prev. Sci..

[b0080] Lee H., Kim Y., Terry J. (2020). Adverse childhood experiences (ACEs) on mental disorders in young adulthood: Latent classes and community violence exposure. Prev. Med..

[b0085] Llabre M.M., Schneiderman N., Gallo L.C., Arguelles W., Daviglus M.L., Franklyn Gonzalez I.I., Isasi C.R., Perreira K., Penedo F.J. (2017). Childhood trauma and adult risk factors and disease in Hispanics/Latinos in the US: Results from the Hispanic Community Health Study/Study of Latinos (HCHS/SOL) Sociocultural Ancillary Study. Psychosom. Med..

[b0090] Marin G., Sabogal F., Marin B.V., Otero-Sabogal R., Perez-Stable E.J. (1987). Development of a short acculturation scale for Hispanics. Hispanic J. Behav. Sci..

[b0095] Maxia D., Anda R.F., Felitti V.J., Dube S.R., Williamson D.F., Thompson T.J., Loo C.M., Giles W.H. (2004). The interrelatedness of multiple forms of childhood abuse, neglect, and household dysfunction. Child Abuse Negl..

[b0100] McCurley J.L., Gutierrez A.P., Bravin J.I., Schneiderman N., Reina S.A., Khambaty T., Castañeda S.F., Smoller S., Daviglus M.L., O’Brien M.J., Carnethon M.R. (2019). Association of social adversity with comorbid diabetes and depression symptoms in the Hispanic Community Health Study/Study of Latinos Sociocultural Ancillary Study: a syndemic framework. Ann. Behav. Med..

[b0105] Mwachofi A., Imai S., Bell R.A. (2020). Adverse childhood experiences and mental health in adulthood: Evidence from North Carolina. J. Affect. Disord..

[b0110] Oh D., Jerman P., Marques S.S., Koita K., Ipsen A., Purewal S., Bucci M. (2018). Systematic review of pediatric health outcomes associated with adverse childhood experiences. Pediatrics.

[b0115] Reiser S.J., McMillan K.A., Wright K.D., Asmundson G.J. (2014). Adverse childhood experiences and health anxiety in adulthood. Child Abuse Negl..

[b0120] Sabogal F., Marín G., Otero-Sabogal R., Marín B.V., Perez-Stable E.J. (1987). Hispanic familism and acculturation: what changes and what doesn't?. Hispanic J. Behav. Sci..

[b0125] Sareen J., Henriksen C.A., Bolton S.L., Afifi T.O., Stein M.B., Asmundson G.J.G. (2013). Adverse childhood experiences in relation to mood and anxiety disorders in a population-based sample of active military personnel. Psychol. Med..

[b0130] Schilling E.A., Aseltine R.H., Gore S. (2007). Adverse childhood experiences and mental health in young adults: a longitudinal survey. BMC Public Health.

[b0135] Shin S.H., McDonald S.E., Conley D. (2018). Patterns of adverse childhood experiences and substance use among young adults: A latent class analysis. Addict. Behav..

[b0140] Tomczyk S., Isensee B., Hanewinkel R. (2016). Latent classes of polysubstance use among adolescents—a systematic review. Drug Alcohol Depend..

[b0145] Zetino Y.L., Galicia B.E., Venta A. (2020). Adverse childhood experiences, resilience, and emotional problems in Latinx immigrant youth. Psychiatry Res..

